# “They forgot about us”: experiences of the COVID-19 pandemic among people deprived of housing in an urban centre in Ontario, Canada

**DOI:** 10.17269/s41997-023-00793-2

**Published:** 2023-08-01

**Authors:** Larkin Lamarche, Eilish Scallan, Orianna Mak, Jillian Howden, Claire Bodkin, Lisa Nussey, Kelly Wolf, Jody Ans, Danielle Delottinville, Tim O’Shea, Robin Lennox

**Affiliations:** 1 Department of Family Medicine, David Braley Health Sciences Centre, Hamilton, ON Canada; 2 School of Kinesiology and Health Science, York University, Toronto, ON Canada; 3 Faculty of Health Sciences, McMaster University, Hamilton, ON, Canada; 4 Hamilton Social Medicine Response Team (HAMSMaRT), Hamilton, ON, Canada; 5 McMaster Midwifery Research Centre, McMaster University, Hamilton, ON, Canada; 6 Keeping Six, Hamilton, ON Canada; 7 Department of Medicine, McMaster University, Hamilton, ON, Canada

**Keywords:** Homelessness, Public health emergency, Pandemic, COVID-19, Sans-abrisme, urgence sanitaire, pandémie, COVID-19

## Abstract

**Objectives:**

People deprived of housing have been disproportionately affected by the COVID-19 pandemic and the public health mitigation measures implemented in response. Emerging evidence has shown the adverse health outcomes experienced by these communities due to SARS-CoV-2 infection; however, the voices of community members themselves have not been widely amplified in the published literature.

**Methods:**

We conducted an interpretive qualitative study. People deprived of housing were involved in study development, recruitment, and data analysis. People deprived of housing or precariously housed were recruited during street outreach from June to July 2020. Participants completed one-on-one semi-structured interviews that were audio-recorded, transcribed, and analyzed thematically.

**Results:**

Twenty-one participants were interviewed. Central to participants’ experiences of the COVID-19 pandemic were descriptions of access to services, in terms of both changes in service availability and the reality of how accessible existing services were to the community, represented by the theme *access*. Four other themes were generated from our analysis and include *feeling and being unheard*, *stripped of dignity*, *I’ve been broken*, and *strength and survival* (with a subtheme, *community care*).

**Conclusion:**

Future emergency response efforts must meaningfully engage people deprived of housing in planning and decision-making in order to minimize adverse impacts of health emergencies and the associated public health responses. There needs to be more careful consideration of the unintended harmful impacts of public health measures implemented in response to pandemics.

## Résumé

**Objectifs:**

Les personnes sans abri ont été démesurément affectées par la pandémie de COVID-19 et par les mesures sanitaires mises en œuvre en réponse à la pandémie. Des données probantes émergentes montrent les résultats sanitaires indésirables éprouvés par ces communautés en raison de l’infection par le SRAS-CoV-2, mais les voix des membres de ces communautés ne sont généralement pas amplifiées dans les articles publiés.

**Méthode:**

Nous avons mené une étude qualitative interprétative. Des personnes sans abri ont été mises à contribution dans l’élaboration de l’étude, le recrutement des participants et l’analyse des données. Des personnes sans abri ou au logement précaire ont été recrutées lors d’activités d’approche dans la rue en juin et juillet 2020. Elles se sont prêtées à des entretiens semi-directifs en tête à tête qui ont été enregistrés, transcrits, puis analysés par thèmes.

**Résultats:**

Vingt et une personnes ont été interviewées. Leurs expériences de la pandémie de COVID-19 ont été fortement axées sur le thème de *l’accès* aux services, tant pour ce qui est des changements dans la disponibilité des services que de l’accessibilité réelle des services existants pour les membres de ces communautés. Quatre autres thèmes sont ressortis de notre analyse : *le sentiment/le fait de ne pas être entendu, d’être dépouillé de sa dignité*, *d’avoir été brisé,* et *la force et la survie* (et un thème secondaire : les soins de proximité).

**Conclusion:**

Les futurs efforts d’intervention d’urgence doivent inclure une véritable collaboration avec les personnes sans abri dans la planification et la prise de décisions afin de réduire les répercussions néfastes des urgences sanitaires et des mesures de santé publique connexes. Il faut examiner plus attentivement les effets pervers des mesures sanitaires mises en œuvre en réponse aux pandémies.

## Introduction

On March 11, 2020, the World Health Organization (WHO) officially declared the COVID-19 pandemic, due to the novel coronavirus (SARS-CoV-2; see WHO interactive timeline: World Health Organization, (n.d.)). Concerns about the impact of the pandemic on people deprived of housing in Canada were raised shortly thereafter (Perri et al., 2020). People deprived of housing, broadly including people who are homeless, houseless, and sleeping rough, and who have insecure or sub-standard accommodation (Springer, 2000), are at higher risk of both acute and chronic diseases, and experience increased disease severity and higher mortality rates than the age-matched general population (Hwang, 2000, 2001). Thus, it is not surprising that people deprived of housing have been identified as a population at increased risk during a pandemic. We know from previous pandemics (SARS, H1N1) that there are unique issues related to serving people deprived of housing. Public health officials identified concerns over communication, infection control, quarantine, and resource allocation after the 2003 SARS outbreak in Toronto (Buccieri & Schiff, 2016; Leung et al., 2008).

People deprived of housing have been disproportionately affected by SARS-CoV-2 infection. In a retrospective cohort study conducted in Ontario, Canada, people deprived of housing had higher SARS-CoV-2 test positivity rates than people with no recent history of homelessness (Richard et al., 2021). They also had higher rates of complicated illness requiring acute care (20 times more likely to experience hospitalization and 10 times more likely to require ICU admission; Richard et al., 2021). In the 21 days following a positive test, the mortality rates were five times higher than for people with no recent history of homelessness (Richard et al., 2021).

COVID-19 pandemic public health strategies have called for individuals to physically distance, isolate, reduce non-essential activities, and adhere to masking and hygiene practices. Though essential for infection prevention and control, these measures pose challenges to people deprived of housing, as they often have little control over their physical and social environments and may rely on congregate settings for survival (Perri et al., 2020).

While mortality- and morbidity-related studies have emerged, we lack studies that describe the pandemic experiences of people deprived of housing. By understanding these unique experiences (in combination with epidemiology evidence), we can better develop strategies and policies, informed by those experiences, so that interventions do not unintentionally harm individuals deprived of housing (Perri et al., 2020). Public Health may be well positioned to integrate all forms of science into policies and interventions. Our research question is: what are the unique experiences of the COVID-19 pandemic for people deprived of housing in Hamilton, Ontario?

## Methods

### Study setting

This study occurred in Hamilton, Ontario, Canada, during June and July of 2020. During this time, according to Public Health Hamilton (n.d.), the 7-day average rate of SARS-CoV-2 was between 5 and 9 per 100,000 in Hamilton. A state of emergency was declared in Ontario in March 2020 and continued throughout data collection. Public health restrictions included closure of all non-essential businesses, public facilities (public libraries, indoor recreational centres), and schools, and limitations on gatherings (see https://www.ontario.ca/laws/regulation/200051).

Members of the Hamilton Social Medicine Response Team (HAMSMaRT) and Keeping Six joined forces to go on street outreach three times per week to listen to people’s needs and challenges and provide basic supplies (food, clean water, hand sanitizer, harm reduction supplies). HAMSMaRT is an organization of health providers (some are also researchers) and community organizers working to integrate clinical practice, critical analysis, and political action in the health and social care systems within Hamilton. Keeping Six is a group of people who use drugs and those who love and care for them, and their mission is to defend the rights, dignity, and humanity of people who use drugs.

### Research team and philosophical underpinnings

The team included members of Keeping Six and HAMSMaRT, with three medical students, who were embedded in outreach activities. Our members have authentic, diverse, and long-standing connections to communities of people deprived of housing, and it is with these connections that we reflect. We orient from a pragmatic worldview driven by understanding and solving practical problems, which are socially, historically, and politically contextualized and align with social justice (Morgan, 2014). We attempted to flatten the usual power imbalances (especially those driven by knowledge and “expertise”; Faulkner, 2017) between researchers and communities being researched, and between clinicians and students.

### Recruitment and participants

Upon institutional research ethics board approval, participants were purposive sampled with diversity in shelter in mind (e.g., people sleeping rough, in shelters, or in pandemic-specific arrangements including hotels and arenas). Potential participants were recruited by staff at drop-in spaces, at clinician encounters, and during street outreach. The medical students helped to recruit and conduct the interviews. Participants were introduced to the study and asked to complete the one-on-one interview “on-the-spot”. Where “on-the-spot” interviews were not possible, the medical students followed up at the drop-in space or next clinician encounter.

All participants were English-speaking and living in Hamilton. Participants were compensated $30. After the 21^st^ interview, it was decided that data richness, depth, diversity, and complexity (Nelson, 2016) were sufficient and recruitment was ended.

### Interview guide

All members helped to develop the interview guide (see Appendix). The interviewers were trained by the first author. The first interview conducted by each interviewer served as a pilot; all were deemed successful based on good interview practices and the richness of the data collected, and were included in analysis.

### Data collection and management

Interviews were conducted in-person using public health guidelines (physical distancing, mask wearing), or over the phone, and audio-recorded. A pseudonym was chosen by each participant (some chose a full name, some just initials, and one chose a single letter). Audio-recordings were professionally transcribed. One interview was excluded from analysis because the information was not grounded in the interview guide.

### Data analysis

For training, the interviewers and the first author independently inductively coded the same interview. They met to discuss codes and initial impressions. All coders felt comfortable to continue coding, and then independently coded the interviews they conducted.

Our process followed Braun and Clark’s (2006, 2019) six phases of reflexive thematic analysis. Specifically, the interviewers read and re-read the transcripts, and relistened to the audio-recordings; generated initial codes which were latent and inductive, grounded in the data; grouped codes together to generate themes; and the first author, interviewers, and the last author reviewed potential themes through team discussions. Our collective meetings involved reflecting with the data in connection to our experiences “on the front line” and what we were seeing, hearing, and feeling in our outreach and clinical work, and historically in Hamilton. Our experiences within the communities provided congruence for the themes. We defined and named themes and confirmed thematic structure with a full-team, consensus meeting. Placing themes within the historical pattern of ignoring, forgetting, and silencing people deprived of housing within the city helped elucidate meaning-making. We also deductively coded the data to specifically look for impacts of the pandemic which informed our interpretation of the data. Finally, we produced this paper. We took an interpretive approach (Finlay, 2021).

### Rigour

We used various techniques to foster rigour (Lincoln & Guba, 1986; Tracy, 2010). Credibility, a measure of truth value, was upheld by using purposive sampling. Half of our research team were made up of members from Keeping Six and HAMSMaRT, and who accompanied these organizations on their outreach activities, to foster our credibility as researchers; this was in addition to our previous engagement with the communities recruited. We used direct quotations to capture participants’ perceptions for authenticity. Trustworthiness was upheld by our interview training process. Also, we practiced reflexivity in analysis meetings, providing depth to the analysis, drawing from our diverse and historical collective of clinical, academic, and experiential experiences (i.e., diverse forms of knowledge; Faulkner, 2017).

## Results

Below we describe five main themes (and one sub-theme): *access*, *feeling and being unheard*, *stripped of dignity*, *I’ve been broken*, and *strength and survival* (*community care*). Central in the data, and presented first, was the theme *access?*; contents of this theme dominated all conversations. The question mark of *access?* visually represents participants’ questions around theoretical as well as material access. We decided to include the question mark in the sections below to disrupt reading this paper, and in so doing, symbolize what participants’ experience of *access?* was: sometimes appropriate and helpful, but at other times disruptive or misplaced. This decision also represents an example of our reflexivity—we also question access. The remaining themes operated around the central theme of *access?* and are detailed below in no particular order.

### Access?

At the most descriptive level, people talked about the types of supports and services (un)available within Hamilton. These included church drop-ins, additional shelter beds, bathrooms, food, water, and harm reduction supplies. This theme also included changes in access to supports and services because of the pandemic. For example, there was recognition of increased shelter beds in the city:

I think it’s really good that they’ve kept [women’s emergency shelter] open. Like it was supposed to be just an overflow shelter during the winter months. The only reason it got extended past even like February, March kind of thing is because of COVID, because they understand like we need to be housed or at least have somewhere to go at night. (R)

However, sometimes changes meant a closure of a space typically accessed pre-pandemic or changes to availability. L described the closures of two favourite places, “I spent quite a bit of time in the gym and that’s closed now. And also at the library, because I love to read, but that is closed now so there’s not a whole lot.” Access to services were also restricted: “Laundry and clothing. Like that’s the biggest support we lost” (R). Some participants described changes to the accessibility of supports and services through the pandemic to date:

In the winter it was really rough because we wouldn’t have a [coffee shop] or anything to go into to use the washroom or even just sit down; you have to go to warming centres, which they didn’t even start opening up until like March, April, when the cold had already gone by. And with [emergency shelter] at the [organization], that doesn’t open until like 10:00PM, I think, so that was really rough having to not be able to go into a warm shelter to sleep at night until 10:00PM or 9:00PM, it wasn’t very logical. (Agatha)

Many participants questioned the access?ibility of services. For example, much of the day-to-day survival relied on opening and closing times, which were subject to frequent changes. R’s comment about time highlights the challenge of trying to navigate what is open:

I try and find out what time it is, because there’s like two people out of like five or six or more people at the campsite that actually have working cell phones, and no electricity to charge said cell phones. If they die on us in the middle of the night then we have like no clue like what time it is...And where I have access to, again, depends on when I wake up.

The accessibility of public health guidelines was also questioned:

I’m aware of some of them [public health guidelines], but I’m sure there are some I’m missing. Like not having real access to the media, or to even like public health, like their website and stuff on a regular basis. (R)

### Feeling and being unheard

This theme name resulted from reflecting on our experiences and knowledge of historical patterns of ignoring, forgetting, and silencing of people deprived of housing in Hamilton; for us, the theme name represents how it feels to be excluded, not considered, overlooked, and disregarded in decisions about your communities, and shows the issue is with the system and not the person. Participants articulated an experience of feeling not included in the decisions affecting their lives and their survival. Participants identified a failure to consider the needs of some members, particularly women and transgender folx deprived of housing. Jen explained, “the women of Hamilton have been forgotten, the single women without children in Hamilton have been forgotten…somebody help them.” CB criticized the exclusion of women from an emergency shelter space opened during the pandemic:

I still don’t necessarily agree with them opening [the centre] as strictly an overflow men’s shelter. I honestly think that they should have opened it up as co-ed and figured out a way to separate the men from the women. But they have 75 beds in there and they’re not even at capacity. Like 75 beds, I think the last I heard that they had like 50 something guys that are in there. So they are not at capacity. The women’s overflow shelter, we have mattresses on the floor about an inch thick.

Some participants discussed the idea of being *unheard* in public health guidelines and the misperception that all members of society would be able to adhere to these with the same ease. As Sam stated:

it’s been almost impossible to adapt because every time we change something in order to try and make ends meet, the rules around it change almost instantly — it feels like every single time we come to a reasonable solution to help ourselves get a little bit of leverage, they immediately pull the lever from out from underneath us.

Participants discussed the impacts of pandemic-related by-laws that did not consider the realities of those deprived of housing, specifically an increase in ticketing for toileting outside or inability to physically distance. CB said:

Stop ticketing us. I myself haven’t been ticketed for anything COVID-19 related, but I know one person that got two tickets for like $750 each within two days. It was for social distancing. It was one of those. And when he told me I just looked at him and I thanked him for letting me know because I was gonna tell one of you guys about it. And, yeah, like, you know, COVID-19 is almost — is basically putting people out on the streets, but the people that are on the streets aren’t able to live on the streets or be on the streets without getting ticketed.

As one participant described the disconnect caused by the lack of community voices in decision-making, “that’s not how we would work” (Andreo).

This theme was closely connected to the theme of *access?* in that the lack of understanding community needs manifested in the (un)availability of supports. This was especially prominent in relation to hygiene facilities and emergency shelters. Many community needs were not considered, like laundry services:

Like everybody makes sure we’re fed, everybody make sure we have water, everybody makes sure we have somewhere to stop in and get out of the heat, but nobody really thinks twice about seeing the same person show up in the same outfit, you know, a week in a row…Like come on, at least give me a washboard. (R)

R articulated a lack of understanding of community needs with the provision of porta-potties for washroom access?:

The porta-potties down outside of [place], great idea, poor execution.... It’s kind of really awkward when you’re like female, especially for someone like me who has a history of like sexual assault. It’s like you drop your shorts or whatever to go pee, and the next thing you know you’ve got this eyeball in the wall. It’s like, ‘I’m out of here.’(R).

Though access to washrooms was appreciated by some, important aspects of hygiene were not addressed. As Crystal said, “there’s things that we need, a bathroom — which they were amazing by putting porta-potties up. But how are you gonna wash your hands?”. Sarah Parker agreed, “There’s no bathrooms that we can really wash our hands in. So we can’t use bathrooms and those outhouses down there, they don’t even have a sink, they don’t even work, and they’re freaking disgusting, and they smell so bad.”

### Stripped of dignity

The theme *stripped of dignity* emerged as participants questioned solutions relating to *access?*. The lack of appropriate hygiene facilities was one example:

It’s taken away our humanity and our dignity. Yeah. Our dignity, the last little pieces of dignity that you have left, yeah, they took that away because they forgot about us. It took them three weeks to put port-o-potties outside after they closed all the bathrooms. So where else are you supposed to go to the bathroom, right? Like that’s your dignity. You know, as a human being, being forced to like pee and poop outside - not cool. (Jen)

Sam talked about interactions with strangers in the city during COVID:

Everybody is being told not to even approach us. And it hit so freaking hard that now, for the most part, when we walk down this street we’ll approach five or six people to ask them a question as basic as the time or if they happen to have a spare cigarette, and we’ll get the stare, and they won’t say a word, as though this thing could be caught just by them talking to us. And that is so freaking hurtful.

Jen described her understanding of the system used to determine suitability for overflow shelter spaces in a hotel, and her experience of having her dignity stripped by the process:

The hotel system that I’m in right now, that’s what it was set up for, like they had good intentions, but the way that they ran them was wrong. It was like a voting committee; it was like if you behaved well, you got in here; if you didn’t it was like they got to vote and you had to put the people up, they had to be nominated. And I mean, it destroyed a lot of people. It did. It destroyed them because your self-worth is tied up in that. And it shouldn’t have been based on merit — it should be based on need. All the women know that every Tuesday they have a meeting where they vote who gets to go into the hotel this week, right. And every Wednesday morning it would be, everybody would wake up and there would just be that sense of dread of knowing that you didn’t get voted in again.

Though the selection process for the overflow shelter spaces was not explicitly shared with women deprived of housing, Jen highlighted the impact that the perceived selection process had on those who were not offered spaces in the hotels, stating, “I’m not sure what they were trying to achieve, but what they did achieve and especially with women, was lowering of self-esteem and self-worth in a traumatic way.”

### I’ve been broken

Sarah Parker’s quotation illuminates the essence of this theme so well that we named the theme after her quotation, indicative of the overall experience of COVID-19, in her response to the full-body check-in: “I’m broken. That’s how I feel; I feel broken…” The full-body check-ins gave space for participants to express any challenges during the pandemic. Jen said, “I am congested emotionally”; Victor felt “numb” and “pretty empty”; Agatha and Victoria “[felt] drained”; Roxanne was “very overwhelmed to the point where [she doesn’t] really feel anything.”

There was also a sense of loneliness, isolation, disconnection, and loss of identity brought on by the pandemic: “Most of the community has been ripped apart. All of the hangout spots have been squashed. And a lot of friends have vanished or been arrested or worse” (Sam). Ryan mentioned, “I feel like I’m isolated in a way. Yeah, just feel kind of isolated, meaning like, you know, there’s like police reinforcement with social distancing and like no group gatherings and stuff like that.”

The theme of *I’ve been broken* was powerfully tied to a feeling of hopelessness for some. As L stated:

...right now hope is not really a luxury I can afford to have. I have always felt that way about homelessness, you know, it’s not just a lack of a roof, it’s sort of like an outlook on life and a mental state. And one of the things about it is not having hope, and you know, knowing that that’s not what you can afford to have. Like most people have hope - like when they finish school, I’m gonna get this great job or something, you know, have those kinds of dreams and hopes, but, you know, poor people can’t afford to have that.

### Strength and survival

Experiences were not only characterized by hardship. The theme *I’ve been broken* must be understood with the theme *survival* (and vice versa), for deeper, nuanced meaning-making of the data. Paired with the physical, emotional, and psychological toll of the pandemic, there was a sense of strength and survival—something that participants always had, drew on, and depended on, and that was a source of pride. Veronica described the day as keeping on task to meet basic needs, “For me right now it’s just keeping on task, having an itinerary every day, trying to check off the items, Maslow’s hierarchy of needs, food, water, shelter, and move up from there.”

At a social level, Jen described how she and her husband (who was housed in the men’s shelter) would steal moments together:

it would be about trying to steal times. Like my husband and I would ride the bus together to spend time together. At night we would like go in the back of a bus where a bus driver wouldn’t yell at us for sitting next to each other and ride the bus together to stay warm and to like spend time together and to just like, you know, physical contact with each other after being married for 15 years.

As mentioned, participants expressed how the pandemic had changed survival among the community. Agatha explained the financial impact, “Panhandling is difficult too because people aren’t making as much money as they were so they’re not willing to toss change at us or whatever…the money situation has never been good for me, but right now it’s even worse because panhandling is not easy.” Sam remarked how “when it first started, there were a couple news channels that were reporting that the highest [COVID-19 case] count was in the homeless population and that it’s found on their signs and in their money and so on and so forth, so don’t approach them by any means whatsoever. And that hit so hard on the panhandlers.” Despite these challenges, there was a sense of strength, “I know I’m doing the best I can. And we’re getting by. We made it through today; we made it through yesterday. I know we’re going to make it through tomorrow even if it is hectic and rough and not always fun.”

### Community care

Community care developed as a sub-theme of *strength and survival*. Threaded throughout the interviews was gratitude and acknowledgement of community organizations, shelters, churches, and complete strangers that in some way made a difference. The types of supports included things like food, shelter, and washrooms, and also emotional support and an overall feeling of collectivism. JW said: “I like how it sort of like worldwide like everybody community coming together and helping each other out that we’re seeing. I hope that continues. That’s what I would hope for.” Veronica expressed how she found gratitude and hope in her interactions with some in the city:

If it wasn’t for people and keeping faith, that smile that I get on the street, I won’t even know what your name is, but thank you, you know. That person that say, ‘Can I give you a cigarette?’ Thank you. For the gentleman at the [bus station] that gave me $7 yesterday and gave me the papers and where to find the thing, he was a bus employee and he’s not even working in a shelter and he tried. It’s not just one organization, we all have to come together.

Similarly, part of community care was the connection among the homeless communities; CB mentioned, “when we’re all homeless, and our family, our street family is our family, no matter how big or small it is. I remember saying to one of my friends I’m like, ‘We have to stick together through this like.’” Jake Wobbler highlighted the feeling of mutual aid, saying “I’m still going on with that caring amongst each other, like just keep it like that.”

## Discussion

This study explored the experiences of the pandemic among individuals deprived of housing in Hamilton. Prominent themes emerged around lack of access?ibility of essential services, lack of direct engagement of those deprived of housing in emergency response planning, threats to dignity, and the ways that people fought to survive and communities that cared for people when systems failed.

While these themes speak specifically to the experiences during the pandemic, they are connected to recurring narratives in research related to the experiences of being deprived of housing more broadly (e.g., Walsh et al., 2016). For example, Hoffman and Coffey (2008) found that descriptions of interactions between service providers and people deprived of housing were commonly expressed in negative terms and contained experiences of objectification and infantilization. As a result, people often opted out of the social service system to maintain a sense of dignity and self-respect.

The changes to service availability and accessibility early in the pandemic response highlight the need to consider the unintended consequences of emergency public health measures, with specific attention to populations that may be disproportionately affected. Mosher (2013) explored the ways in which pandemic plans fail to account for the needs of those facing social exclusion, despite the reality that those with the highest levels of social and economic disadvantage bear the brunt of pandemic illness and mortality. Mosher highlights the ways that voice and participation from socially excluded groups have been made invisible by governments during times of pandemic and in pandemic planning (e.g., SARS, H1N1). Our findings point to an enduring lack of understanding of the lived/living experiences and needs of people deprived of housing in pandemic planning and response from the public health and homelessness sector decision makers. Our own experiences tell us that some unintended impacts could have been mitigated if there were greater (and genuine) engagement of people deprived of housing in the planning process.

One of the least active forms of participation is consultation (Sprague et al., 2019); using approaches that involve more active participation (participatory-based co-design, co-development) is necessary to meaningfully engage with communities, particularly those that are stigmatized, in order to dismantle power relationships and balance the scales of equity (Sprague et al., 2019). This kind of engagement needs to be taken up outside of times of crisis, to ensure trust and efficient response during emergencies. However, the context we are currently working in is already in crisis. Across Canada, we have an affordable housing crisis (Schwan et al., 2021) and toxic supply crisis (Belzak & Halverson, 2018). In a recent Superior Court decision, because there are not enough accessible shelter beds, Kitchener, Ontario, Canada, cannot evict (through a bylaw enforcement) members of an encampment because it infringes upon their Canadian Charter of Rights and Freedoms (The Regional Municipality of Waterloo, 2023). This kind of engagement, which ensures people’s needs are actually met by the systems serving them, is not only ethical, but also practical and efficacious; when done properly, it can result in services that attend to the needs of communities, as they define them (Norman et al., 2015). As a local advocate and outreach worker with lived experience of homelessness wrote, “If you want to see a change, then allow me to lead the way. I can show you in an hour what it takes you years to figure out without me” (McIlveen, 2021). Advocating for *working with* approaches is nothing new; such approaches have been suggested by many (e.g., Walsh et al., 2016).

Gender-specific concerns identified by study participants mirror the systemic barriers facing women and gender-diverse individuals. The Pan-Canadian Women’s Housing and Homelessness Survey in 2021 identified that only 13% of emergency shelter beds in Canada are dedicated to women (Schwan et al., 2021), compared to 36% of respondents in a 2018 national point-in-time count identifying as female (Employment and Social Development of Canada, 2019). In Hamilton, there has been a significant increase in the proportion of women experiencing homelessness—from 32% in 2018 to 53% in 2022—illustrating the increasing need for shelters for women (City of Hamilton’s Housing Services Division, 2022). Though 38% of emergency shelter spaces are designated as co-ed or open to all genders, many women will not access? shared spaces due to prior experiences of violence (Schwan et al., 2021). Many female-identifying study participants discussed the disparities in emergency shelter spaces, and a lack of consideration for the needs of individuals who have experienced sexual or gender-based violence in the delivery of essential services (e.g., co-ed porta-potties located in public spaces). Our findings reinforce the need to ensure a gender equity lens is applied in the process of pandemic planning.

### Strengths and limitations

Our connections with the local communities helped enrich the interpretation of the data. Our data collection was in English, and did not capture the experiences of people who do not speak English and would be more likely to exclude racialized migrants. These experiences are drawn from an urban city and may not represent experiences in other settings across Canada, or internationally. We also did not explicitly collect demographic information; we used trauma-informed practices, acknowledging that collecting demographic information can do harm, especially if it is not central to a purpose (i.e., it can “out” somebody; Isobel, 2021). The experiences shared by participants captured a snapshot during the pandemic. The nature of the pandemic has dramatically changed since data collection (e.g., prevalence of variants, changes to public health restrictions, vaccine availability).

## Conclusion

There were unique impacts in the initial pandemic response effort, possibly due to an incomplete understanding of specific survival needs of people deprived of housing. Our findings suggest that direct involvement of community members in pandemic response planning may yield more effective, community-focused solutions. With a principle of community engagement at the forefront, it will be possible to develop policies that centre those who have traditionally been forgotten.

## Contributions to knowledge

What does this study add to existing knowledge?

- Adds qualitative evidence about experiences of those deprived of housing during the pandemic.
- Highlights unintended consequences of public health measures among people deprived of housing, with emphasis of gender-specific concerns.
- Adds to growing calls for community-engaged approaches to public health measures and solutions.

What are the key implications for public health interventions, practice, or policy?

- Interventions, practices, and policies can do unintentional harm; we argue for widespread uptake of, and long-term commitment to, community-engaged policy development and practice (beyond simple consultation).

## Data Availability

Reasonable requests for data and study materials available upon email request to the corresponding author.

## Appendix: Interview guide

1. We first want to do a full body check in and ask how is your head? How is your heart? How is your body?
2. Tell me what a typical day for you looks like right now during the pandemic?
3. How has the COVID-19 pandemic impacted your community?
4. What are your thoughts / beliefs about all that has happened with the COVID-19 pandemic?
5. What are you most hopeful for right now?
6. What are you most fearful of right now?
7. How do you feel like the needs of people who use drugs and/or those experiencing homelessness have been accounted for in the pandemic response planning?
  a Are there ways that your needs are not being met right now?
  b Are there ways your needs could be better met right now?
  c If you could tell decision makers in Hamilton one thing, what would it be?
8. Have there been any changes to the services or supports available to you during the COVID-19 pandemic?
  a If so, which services or supports have been affected? How have these impacted your life or well-being?
9. Can you tell me about your experiences with public health guidelines?
  In these first interviews, challenges in following public health guidelines were articulated. We subsequently added an interview question that addressed this directly, as we felt the information helped us understand unique experiences of the pandemic.
